# Serum cystatin C, impaired kidney function, and geriatric depressive symptoms among older people living in a rural area: a population-based study

**DOI:** 10.1186/s12877-018-0957-2

**Published:** 2018-11-06

**Authors:** Ling Wu, Zhongrui Yan, Hui Jiang, Huaimei Xing, Haohao Li, Chengxuan Qiu

**Affiliations:** 1 Cheeloo College of Medicine, Shandong University, Jinan, Shandong China; 2 Department of Neurology, Jining No. 1 People’s Hospital, Jiankang Road 6, Jining, 272111 Shandong China; 3 Xing Long Zhuang Hospital, Shandong Yankuang Group, Jining, Shandong China; 4 Department of Neurology, Shandong Provincial Hospital, Jinan, Shandong China; 5 Aging Research Center, Department of Neurobiology, Care Sciences and Society, Karolinska Institutet-Stockholm University, Widerströmska Huset, Tomtebodavägen 18A, 171 65 Solna, Sweden

**Keywords:** Aging, Cystatin C, Kidney function, Depressive symptoms, Cohort study, China

## Abstract

**Background:**

The relationship between kidney function and depressive symptoms among elderly people has been rarely investigated in settings of the general population. The aim of our study was to examine the association of serum cystatin C (cysC) and impaired kidney function with geriatric depressive symptoms among older people living in a rural community in China.

**Methods:**

This population-based cohort study included 1440 individuals (age ≥ 60 years) who were recruited for the Confucius Hometown Aging Project in 2010–2011; of the 1124 persons who were free of depressive symptoms, 669 (59.5%) were re-examined in 2014–2016. At baseline, data on demographics, lifestyle factors, health conditions, and medical history were collected through interviews, clinical examinations, and laboratory tests. We defined impaired kidney function as the cystatin C-based estimated glomerular filtration rate (eGFR_cysC_) < 60 ml/min/1.73 m^2^, and depressive symptoms as a score ≥ 5 on the 15-item Geriatric Depression Scale. Data were analyzed using multiple logistic and Cox proportional-hazards models.

**Results:**

Of the 1440 participants, 316 (21.9%) were defined to have geriatric depressive symptoms at baseline. Serum cysC levels of 1.01–1.25 and > 1.25 mg/L (vs. ≤1.00 mg/L) were associated with a multiple-adjusted odds ratio (OR) of 1.41 (95% CI 1.01–1.97) and 3.20 (2.32–4.41), respectively, for having geriatric depressive symptoms (P_trend_ < 0.001). Of the 669 people who were free of depressive symptoms at baseline, 157 had incident depressive symptoms at the follow-up examination. The multiple-adjusted hazard ratio (HR) for incident depressive symptoms were 2.16 (95% CI 1.43–3.27) for serum cysC > 1.25 mg/L (vs. < 1.00 mg/L). Impaired kidney function was cross-sectionally (multiple-adjusted OR = 2.95; 95% CI 2.22–3.92) and longitudinally (multiple-adjusted HR 1.54; 95% CI 1.03–2.30) associated with an increased risk of geriatric depressive symptoms.

**Conclusion:**

Elevated serum cysC levels and impaired kidney function are associated with an increased risk of geriatric depressive symptoms among Chinese older people living in a rural community.

## Background

As population ages, geriatric depression has become a public health concern. Depression in older people is related not only to poor health-related quality of life [1], but also to morbidity, mortality, and even suicide [2]. The Global Burden of Disease 2010 study identified that depressive disorder was a leading cause of years lived with disability (YLDs), and that major depressive disorder accounted for 8.2% of global YLDs [3]. In China, the meta-analyses showed that the prevalence of depression ranges from ~ 20% to ~ 30% among older adults living in communities, with the pooled prevalence being ~ 23% [4, 5]. However, depression has been both underdiagnosed and undertreated in primary care settings owing to common co-occurrence with other geriatric conditions in older adults [6]. Therefore, it is particularly important to identify risk factors related to geriatric depression for possible intervention.

Chronic kidney disease or decreased renal function is increasingly common as people age [7]. In US, chronic kidney disease, defined as an estimated glomerular filtration rate (eGFR) < 60 mL/min/1.73 m^2^, affected nearly one-third of community-dwelling adults aged over 70 years [8]. However, only a few population-based studies have so far examined the relationship between kidney function and depression in older adults, with mixed results. For instance, a cross-sectional study of Chinese older people aged 70–84 years indicated that eGFR < 60 mL/min/1.73 m^2^ was associated with depressive symptoms [9], whereas the Singapore Longitudinal Aging Study found no association between the serum creatinine-based eGFR and depressive symptoms (defined as the 15-item Geriatric Depression Scale [GDS-15] score ≥ 5) [10], and the cross-sectional survey of people over 60 years of age with diabetes in US suggested that only very low eGFR (< 29 mL/min/1.73 m^2^) was associated with an increased risk of depressive symptoms [11]. However, the population-based longitudinal studies of the relationship between measures of kidney function and geriatric depressive symptoms in older adults are still lacking, especially among Chinese elderly people living in rural areas.

Therefore, we hypothesize that increased serum cystatin C or impaired kidney function is associated with an increased risk of geriatric depressive symptoms in older people. We sought to test this hypothesis in this population-based cohort study of older adults who were living in a rural community in China.

## Methods

### Study participants

**Fig. 1:**
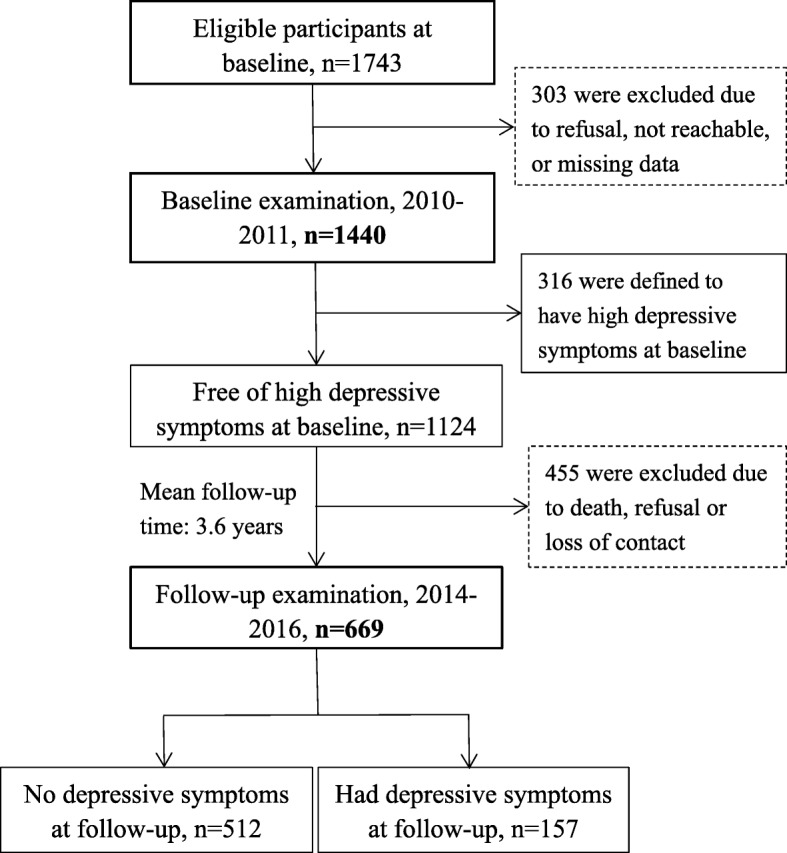
Flowchart of the study participants, 2010–2011 to 2014–2016

This is a population-based cohort study. Study participants were derived from the Confucius Hometown Aging Project (CHAP), as fully described elsewhere [12]. Briefly, CHAP was aimed at exploring the role of cardiovascular risk factors and atherosclerotic mechanisms in aging and health. In the current study, we sought to specifically examine the associations of baseline serum cysC levels and impaired kidney function with depressive symptoms both at the baseline (cross-sectional association) and the follow-up (longitudinal association) examinations. At baseline (2010–2011), 1440 (82.6%) of the 1743 eligible participants who were aged ≥60 years and were living in the Xing Long Zhuang community nearby Qufu city (the hometown of Confucius) in Shandong, China were examined for CHAP; of these, 316 persons were defined to have depressive symptoms (GDS-15 score ≥ 5). In 2014–2016, we carried out the second wave of assessment in the same community for residents who were aged ≥60 years in June 2014, following a procedure similar to that of the baseline survey. In total, 669 (59.5%) of the 1124 persons who were free of depressive symptoms at baseline underwent the follow-up examination. Figure 1 shows the flowchart of the study participants from baseline to the follow-up assessments.

Data collection for all phases of the CHAP study was approved by the Ethics Committee at the Jining No. 1 People’s Hospital of Jining Medical University, Shandong, China. Written informed consent was obtained from all participants, or in the case of cognitively impaired persons, from a proxy (usually next-of-kin).

### Data collection and definition

At baseline, data were collected by trained physicians through face-to-face interviews, clinical examination, and laboratory tests, as previously described [12]. We collected data on demographics, lifestyle factors (e.g. smoking and alcohol consumption), health conditions and health history (e.g. hypertension, diabetes, heart disease, stroke, cataract, chronic obstructive pulmonary disease (COPD), arthritis, nephritis, and tumor), and use of medications (e.g., antihypertensive agents and blood glucose-lowering drugs) following a structured questionnaire. Arterial blood pressure was measured twice in the sitting position after resting for at least 5 min, and the mean value of the two measurements was used for analysis. Peripheral blood samples were taken after an overnight fast, and fasting plasma glucose (FPG), serum cystatin C (cysC), high-density lipoprotein (HDL), low-density lipoprotein (LDL), and creatinine were analyzed using the enzymatic methods by an Automatic Biochemistry Analyzer (Olympus AU-400, Japan).

Body mass index (BMI) was calculated as the measured body weight in kilograms divided by the square of height in meters (kg/m^2^). Smoking status was divided into never and ever (current or former) smoking. Alcohol consumption was assessed based on the frequency and amount of alcohol intake in a typical day and was dichotomized into yes vs. no. Hypertension was defined as blood pressure ≥ 140/90 mmHg or currently using antihypertensive drugs [13]. Diabetes was defined as having a self-reported history of physician’s diagnosis of diabetes, FPG ≥7.0 mmol/L or current use of hypoglycemic agents or insulin injection [14]. Heart disease, stroke, cataract, COPD, nephritis, and tumor were ascertained through clinical examination, electrocardiogram test, or self-reported physician’s diagnosis or currently taking relevant medications.

Global cognitive function was assessed using the validated Chinese version of the Mini-Mental State Examination (MMSE) [15]. Cognitive impairment was defined according to the education-based cutoff scores on MMSE [16], i.e., an MMSE score ≤ 17 for persons without formal schooling, ≤20 for those with 1–6 years of education, and ≤ 24 for those with ≥7 years of education. Physical functioning was assessed using the Katz’s basic activities of daily living (ADL) [17], which involves six self-care activities of bathing, dressing, toileting, continence, transferring, and self-feeding. Participants who were not able to carry out at least one of the six tasks were considered to have disability in basic ADL.

### Kidney function

We calculated the eGFR according to the following equations based on serum cysC (eGFR_cysC_) [18]: eGFR_cysC_ = 133× (cysC/0.8)^− 0.499^ × 0.996^age^ [× 0.932 if female] (if cysC ≤0.8 mg/L); eGFR_cysC_ = 133 × (cysC/0.8)^− 1.328^ × 0.996^age^ [× 0.932 if female] (if cysC > 0.8 mg/L). Impaired kidney function was defined as eGFR_cysC_ < 60 ml/min/1.73 m^2^ [19].

### Geriatric depressive symptoms

The GDS-15 tool was used to assess the presence of geriatric depressive symptoms at both baseline and follow-up examinations. We considered depressive symptoms to be present if a GDS-15 score ≥ 5, a cut-off that has been widely used for screening depression in older adults, with fairly high sensitivity and specificity (ranging from ~ 75% to ~ 95%) when evaluated against the clinical criteria (e.g., DSM-IV criteria) [10, 20–22].

### Statistical analysis

Baseline characteristics of study participants by having depressive symptoms at baseline were compared using t test for continuous variables and χ^2^ test for categorical variables. We categorized the serum cysC level into ≤1.0, 1.01–1.25, and > 1.25 mg/L [23]. We examined both cross-sectional and longitudinal associations of serum cysC and impaired kidney function with geriatric depressive symptoms. For the cross-sectional association, we used multiple logistic regression model to estimate the odds ratio (OR) and 95% confidence interval (CI) of having geriatric depressive symptoms associated with baseline serum cysC levels and impaired kidney function. To explore the longitudinal association, Cox proportional-hazards models were constructed in participants who were free of depressive symptoms at baseline to estimate the hazard ratio (HR) and 95% CI of having depressive symptoms at the follow-up associated with serum cysC levels and impaired kidney function at baseline. In the Cox models, the follow-up time from the date of baseline assessment to the date of follow-up examination was used as the time scale. For both cross-sectional and longitudinal associations, we reported results from three models, in which we controlled for different factors that potentially confounded the examined associations: model 1 was controlled for age, gender, and education (in years); model 2 was additionally controlled for alcohol consumption and history of chronic diseases (e.g. hypertension, heart disease, stroke, cataract, nephritis, arthritis, COPD); and model 3 was further controlled for MMSE score and ADL-disability. We considered *p* < 0.05 (α) for a two-tailed test to be statistically significant. IBM SPSS Statistics 19.0 for Windows (Armonk, NY: IBM Corp.) was used for all analyses.

## Results

At baseline, the mean age of the 1440 participants was 68.5 years (SD, 4.9), and 60% were women. Of them, 316 had geriatric depressive symptoms, which resulted in the overall prevalence of 21.9%, with the prevalence being higher in women than in men (24.8% vs. 17.7%, *p* = 0.002). Compared with people without geriatric depressive symptoms, those with depressive symptoms were older and had a higher prevalence of chronic health conditions (e.g., hypertension, heart disease, stroke, COPD, cataract, arthritis, and ADL-disability) (Table 1). Furthermore, people with depressive symptoms had a higher level of LDL, serum cysC, and creatinine, but a lower level of HDL, eGFR_cysC_, and MMSE score than those without depressive symptoms. The two groups had no significant difference in educational level, BMI, smoking, diabetes, nephritis, and tumor (Table 1).

**Table 1:** Baseline characteristics of study participants according to depressive symptoms at baseline

Characteristics	Total sample	Depressive symptoms		
	(*n* = 1440)	No (*n* = 1124)	Yes (*n* = 316)	*p*-value
Female, n (%)	864 (60.0)	650 (75.2)	214 (24.8)	0.002
Age (years), mean (SD)	68.5 (4.9)	68.3 (4.9)	69.4 (5.1)	0.001
Education (years), mean (SD)	3.9 (3.4)	4.0 (3.4)	3.7 (3.3)	0.180
BMI (kg/m^2^), mean (SD)	26.4 (8.1)	26.5 (8.9)	26.2 (4.2)	0.599
Current smoking^a^, n (%)	206 (14.3)	162 (14.4)	44 (13.9)	0.970
Alcohol consumption^a^, n (%)	299 (20.8)	247 (22.0)	52 (16.5)	0.018
Hypertension, n (%)	768 (53.3)	573 (51.0)	195 (61.7)	0.001
Diabetes, n (%)	322 (22.4)	246 (21.9)	76 (24.1)	0.415
Heart disease^a^, n (%)	486 (33.8)	357 (31.8)	129 (40.8)	0.009
Stroke^a^, n (%)	124 (8.6)	86 (7.7)	38 (12.0)	0.044
Cataract, n (%)	200 (13.9)	145 (12.9)	55 (17.4)	0.041
COPD^a^, n (%)	199 (13.8)	142 (12.6)	57 (18.0)	0.029
Arthritis^a^, n (%)	518 (36.0)	384 (34.2)	134 (42.4)	0.015
Nephritis^a^, n (%)	69 (4.8)	52 (4.6)	17 (5.4)	0.763
Tumor, n (%)	51 (3.5)	37 (3.3)	14 (4.4)	0.333
cysC (mg/L), mean (SD)	0.99 (0.29)	0.97 (0.29)	1.09 (0.26)	< 0.001
HDL (mmol/L), mean (SD)	1.49 (0.44)	1.50 (0.42)	1.44 (0.49)	0.023
LDL (mmol/L), mean (SD)	2.69 (0.85)	2.67 (0.85)	2.78 (0.87)	0.045
Creatinine (μmol/L), mean (SD)	70.5 (21.8)	69.7 (22.8)	73.3 (17.7)	0.010
eGFR (ml/min/1.73 m^2^), mean (SD)	81.3 (30.6)	84.2 (31.7)	71.2 (23.4)	< 0.001
MMSE score, mean (SD)	26.2 (4.5)	26.4 (4.4)	25.5 (4.8)	0.002
ADL-disability, n (%)	32 (2.2)	12 (1.1)	20 (6.3)	< 0.001

*Abbreviations*: *BMI* body mass index, *COPD* chronic obstructive pulmonary disease, *cysC* cystatin C, *HDL* high-density lipoprotein, *LDL* low-density lipoprotein, *eGFR* estimated glomerular filtration rate, *MMSE* mini-mental state examination, *ADL* activities of daily living

^a^Numbers of subjects with missing values were 5 for smoking, 11 for alcohol consumption, 1 for stroke, 3 for heart disease, 4 for COPD, 2 for arthritis, and 3 for nephritis. In the subsequent analyses, subjects with missing values were placed in the non-exposure group

For the baseline cross-sectional relationship, compared to people with serum cysC level of ≤1.00 mg/l, those with levels of 1.01–1.25 and > 1.25 mg/l had an increased likelihood of having geriatric depressive symptoms, with the multiple-adjusted OR (95% CI) being 1.41 (1.01–1.97) and 3.20 (2.32–4.41), respectively (*p* for linear trend < 0.001). Impaired kidney function (eGFR_cysC_ < 60 ml/min/1.73 m^2^) was significantly associated with an increased likelihood of having depressive symptoms (multiple-adjusted OR = 2.95; 95% CI 2.22–3.92) (Table 2).

**Table 2:** Cross-sectional associations of serum cystatin C and impaired kidney function with prevalent geriatric depressive symptoms (*n* = 1440)

Serum cystatin C or impaired kidney function	N/n	Odds ratio (95% confidence interval)		
		Model 1^a^	Model 2^a^	Model 3^a^
Cystatin C, mg/L				
≤ 1.00	768/119	1.00 (reference)	1.00 (reference)	1.00 (reference)
1.01–1.25	360/79	1.43 (1.03–1.98)	1.40 (1.004–1.95)	1.41 (1.009–1.97)
> 1.25	312/118	3.25 (2.39–4.42)	3.30 (2.40–4.50)	3.20 (2.32–4.41)
P for trend		< 0.001	< 0.001	< 0.001
Impaired kidney function				
No	1070/176	1.00 (reference)	1.00 (reference)	1.00 (reference)
Yes	370/140	3.07 (2.34–4.03)	3.03 (2.30–4.01)	2.95 (2.22–3.92)

N/n indicates number of subjects/number of persons with depressive symptoms

*Abbreviations*: *MMSE* mini-mental state examination, *ADL* activities of daily living

^a^Model 1 was adjusted for age, sex, and education; model 2 was additionally adjusted for alcohol consumption, hypertension, stroke, heart disease, cataract, chronic obstructive pulmonary disease, nephritis, and arthritis; and in model 3, MMSE score and ADL-disability were added to model 2

At an average 3.6 years (SD, 0.62) of follow-up, 157 out of the 669 persons who had no depressive symptoms at baseline were ascertained to have incident geriatric depressive symptoms. People who developed incident depressive symptoms at the follow-up were more likely to be female and to have lower education than those who did not. Cox regression analysis suggested that having an elevated serum cysC level (> 1.25 vs. < 1.00 mg/L) was significantly associated with an increased HR of having incident depressive symptoms, even in model 3 when controlling for multiple potential confounders (HR = 2.16, 95% CI 1.43–3.27) (*p* for linear trend < 0.001) (Table 3). Impaired kidney function at baseline was significantly associated with a 54% increased risk of incident geriatric depressive symptoms detected at the follow-up assessment (multiple-adjusted HR 1.54; 95% CI 1.03–2.30) (Table 3).

**Table 3:** Longitudinal associations of serum cystatin C and impaired kidney function with incident geriatric depressive symptoms (*n* = 669)

Serum cystatin C or impaired kidney function	N/n	Hazard ratio (95% confidence interval)		
		Model 1^a^	Model 2^a^	Model 3^a^
Cystatin C, mg/L				
≤ 1.00	388/78	1.00 (reference)	1.00 (reference)	1.00 (reference)
1.01–1.25	175/40	1.11 (0.75–1.65)	1.22 (0.82–1.81)	1.21 (0.81–1.80)
> 1.25	106/39	2.20 (1.48–3.27)	2.23 (1.48–3.34)	2.16 (1.43–3.27)
*p* for linear trend		0.001	0.001	0.001
Impaired kidney function				
No	544/122	1.00 (reference)	1.00 (reference)	1.00 (reference)
Yes	125/35	1.57 (1.06–2.32)	1.57 (1.05–2.34)	1.54 (1.03–2.30)

N/n indicates number of subjects/number of persons with depressive symptoms

*Abbreviations*: *MMSE* mini-mental state examination, *ADL* activities of daily living

^a^Model 1 was adjusted for age, sex, and education; model 2 was additionally adjusted for alcohol consumption, hypertension, stroke, heart disease, cataract, chronic obstructive pulmonary disease, nephritis, and arthritis; and in model 3, MMSE score and ADL-disability were added to model 2

## Discussion

In this community-based cohort study, we found that geriatric depressive symptoms affected more than one-fifth of Chinese older adults who were living in a rural area. In addition, both the cross-sectional and longitudinal data suggested that higher levels of serum cysC were associated with an increased risk of having geriatric depressive symptoms in a dose-response manner, even after adjusting for multiple potential confounders, including sociodemographic factors, lifestyles, cardiovascular disorders, cognitive function, and physical disability. Finally, impaired kidney function, when assessed based on eGFR_cysC_, was associated with both prevalent and incident geriatric depressive symptoms, independent of multiple confounders. This study suggests that high serum cysC levels and impaired kidney function may be risk factors for depressive symptoms in geriatric populations.

The relationship between renal function and depression among older adults has rarely been explored so far in the general population settings. The cross-sectional data from the Maastricht Study of older adults indicated that albuminuria was associated with incidence of depressive symptoms, whereas the reduced eGFR based on creatinine and cystatin C (eGFR_Cr-cysC_) was not associated with minor or major depressive episodes [24]. The follow-up data from the US Health, Aging and Body Composition Study of community-dwelling older adults suggested that an elevated serum cysC level was associated with a 2-fold increased risk of depression, but impaired renal function, assessed based on eGFR_Cr-cysC_ < 60 ml/min/1.73 m^2^, was not related to depression [25]. Of note, some of the previous studies that do not show an association between reduced eGFR and depressive symptoms in older adults have used either serum creatinine or a combination of serum creatinine and cysC to assess eGFR [10, 24, 25]. Because creatinine excretion is dependent on age, muscle mass, and nutritional status, especially among older adults, serum cysC has been considered to be more accurate and more sensitive in assessing renal function [26, 27]. Indeed, we did not find any association between serum creatinine and depressive symptoms (data not shown). When estimating the eGFR based on serum cysC, we found that reduced eGFR_cysC_ or impaired kidney function was associated both cross-sectionally and longitudinally with an increased risk of having geriatric depressive symptoms among Chinese older adults. This is consistent with a cross-sectional study among Chinese older people (age 70–84 years), in which impaired kidney function was associated with a 1.71-fold increased likelihood of having depressive symptoms (GDS-15 score ≥ 5) [9].

The biological and pathological mechanisms linking serum cysC and impaired kidney function with geriatric depressive symptoms are not fully understood, but multiple pathways are supposed to be involved. First, impaired kidney function or reduced eGFR_cysC_ due to glomerular small vessel disease has been correlated with subclinical cerebral microvascular disease in older adults [28, 29]. Further, cerebral microvascular dysfunction and diseases in brain regions involved in mood regulation were associated with late-life depression (“vascular depression”) [30, 31]. Thus, impaired kidney function or reduced eGFR might be linked with late-life depressive symptoms through cerebral small vessel disease. Second, serum cysC, as a cysteine protease inhibitor, affects the migration of neutrophils and involves the inflammatory process [32, 33], which may impair function of the brain-serotonin system and stimulate the activation of the hypothalamus-pituitary-adrenal axis to cause depressive symptoms through inflammation pathway [34]. In addition, the population-based studies of older adults have linked inflammatory markers (e.g., C-reactive protein, IL-6, and TNF-α receptor 1) with elevated serum cysC or impaired kidney function defined by eGFR_cysC_ [35, 36]. The meta-analysis of population-based studies also supported an association of several inflammatory markers with depression in older adults [37], suggesting the involvement of inflammation in the development of depression. Thus, inflammatory mechanisms may mediate the relationship between serum cysC and depressive symptoms. Of note, our data showed that the association of high serum cysC and impaired kidney function with depressive symptoms was present independent of the inflammation-related diseases such as atherosclerotic disorders, arthritis, and cognitive and functional impairment, suggesting that additional pathways may be involved in linking high serum cysC with geriatric depressive symptoms.

Our cohort study involved both cross-sectional and longitudinal data of older adults who were living in a rural area in China. Moreover, we collected comprehensive data (e.g., demographics, lifestyles, health history, and cognitive and physical functioning) following a standard approach. Thus, we were able to control for a broad range of potential confounding factors. However, our study also has limitations. First, we used GDS-15 to assess geriatric depressive symptoms instead of a clinical diagnosis of depression, although the GDS-15 cut-off score ≥ 5, as a widely screening instrument for major depressive disorders in older adults, did show high sensitivity and specificity in geriatric populations [10, 21, 22]. Second, we used the cysC-based eGFR as an approximation of glomerular filtration rate instead of a direct measurement, although eGFR has been widely used to define kidney function. Finally, the study population was derived from a single rural community in Eastern China, where people had relatively low education and low socioeconomic position. Thus, caution is needed when generalizing our results to other populations.

## Conclusion

This population-based cohort study showed that depressive symptoms were common among Chinese older adults living in a rural community and that high serum cysC levels and impaired kidney function (assessed using serum cysC-based eGFR) were associated with an increased risk of having geriatric depressive symptoms. While our findings warrant further confirmation in other populations, this study suggests that psychological interventions among older adults with impaired kidney function may help reduce the risk of geriatric depression.

## Abbreviations

ADL: Activities of daily living
BMI: Body mass index
CHAP: The Confucius Hometown Aging Project
CI: Confidence interval
COPD: Chronic obstructive pulmonary disease
CysC: Cystatin C
eGFR_cysC_: Cystatin C-based estimated glomerular filtration rate
FPG: Fasting plasma glucose
GDS-15: The 15-item Geriatric Depression Scale
HDL: High-density lipoprotein
HR: Hazard ratio
LDL: Low-density lipoprotein
MMSE: Mini-Mental State Examination
OR: Odds ratio
